# Genetic linkage analysis supports the presence of two susceptibility loci for alcoholism and heavy drinking on chromosome 1p22.1-11.2 and 1q21.3-24.2

**DOI:** 10.1186/1471-2156-6-11

**Published:** 2005-03-01

**Authors:** Irene Guerrini, Christopher CH Cook, Wendy Kest, Audrey Devitgh, Andrew McQuillin, David Curtis, Hugh MD Gurling

**Affiliations:** 1 Molecular Psychiatry Laboratory, Windeyer Institute for Medical Sciences, Department of Mental Health Sciences, Royal Free and University College London Medical School, 46 Cleveland Street, London, W1T 4JF, UK; 2 St Chad's College, 18 North Bailey, Durham, DH1 3RH, UK; 3 Academic Department of Psychiatry, St Bartholomew's and the Royal London School of Medicine and Dentistry, E1 1BB, London, UK

## Abstract

**Background:**

In order to confirm a previous finding of linkage to alcoholism on chromosome 1 we have carried out a genetic linkage study.

**Methods:**

DNA from eighteen families, densely affected by alcoholism, was used to genotype a set of polymorphic microsatellite markers at loci approximately 10 centimorgans apart spanning the short arm and part of the long arm of chromosome 1. Linkage analyses were performed using the classical lod score and a model-free method. Three different definitions of affection status were defined, these were 1. Heavy Drinking (HD) where affected subjects drank more than the Royal College of Psychiatrists recommended weekly amount. 2. The Research Diagnostic Criteria for alcoholism (RDCA) 3. Alcohol Dependence Syndrome (ADS) as defined by Edwards and Gross (1976) and now incorporated into ICD10 and DSMIV.

**Results:**

Linkage analyses with the markers D1S1588, D1S2134, D1S1675 covering the cytogenetic region 1p22.1-11.2 all gave positive two point and multipoint lods with a maximum lod of 1.8 at D1S1588 (1p22.1) for the RDCA definition of alcoholism. Another lod of 1.8 was found with D1S1653 in the region 1q21.3-24.2 using the HD affection model.

**Conclusion:**

These results both support the presence of linkage in the 1p22.1-11.2 region which was previously implicated by the USA Collaborative Study of the Genetics of Alcoholism (COGA) study and also suggest the presence of another susceptibility locus at 1q21.3-24.2.

## Background

Epidemiological studies report that alcoholism as defined by the Research Diagnostic Criteria (RDC) affects almost 10–15% of the general population in the USA and 1–5% in Europe [1]. Drinking behaviour is clearly partly determined by cultural and psychological factors, but genetic factors also play an important part as shown by family, twin and adoption studies [2]. The overall heritability for alcoholism has been estimated to be around 50% to 60% [3]. However there are almost certainly multiple aetiological subtypes of alcoholism, which will eventually be shown to have heterogeneous genetic and cultural components [2,4]. Many genetic association studies of alcoholism have sought to identify candidate susceptibility genes, but few linkage studies have been undertaken so far. Two genome-wide linkage studies have been performed on US populations. One was carried out on a well defined population of south western USA American Indians [5] and the other on a large sample of families by the Collaborative Study of the Genetics of Alcoholism (COGA) [6,7]. In the native American Indian study two chromosomal regions provided suggestive evidence for linkage. One was on chromosome 11p close to the DRD4 dopamine receptor and the tyrosine hydroxylase genes and the other on chromosome 4p near the β1 GABA receptor gene. Three loci in the ADH cluster on chromosome 4 also gave evidence of linkage on two point but not multi-point linkage analyses [5]. On the other hand the COGA study, the multi-point linkage analysis provided suggestive evidence of linkage on chromosome 1 and 7 with more modest evidence for a locus on chromosome 2. In addition, there was suggestive evidence for a protective locus on chromosome 4 near the ADH gene cluster [6]. This study [6] implicated chromosome 1 in two distinct regions on 1p21-35. Two-point analysis of affected sib-pairs showed a significant increase of allele sharing for two adjacent markers D1S532 and D1S1588. The multipoint linkage analysis reported a lod of 2.93 for D1S1588. A second region near D1S224, 60 cM apart from the first locus, had a multipoint lod score of 1.65. In the COGA replication set of families [7], linkage near the marker D1S224 with a maximum multipoint lod score of 1.6 was reported. In the combined COGA sample a LOD score of 2.6 was reported near markers D1S2614 and D1S1588. Re-analysis of COGA data [8] for linkage to an alcoholism-related phenotype consisting of alcoholism and depression in the combined COGA samples found a maximum lod of 5.12 near the markers D1S1648 and D1S1588. Furthermore, the region on chromosome 1p near D1S1588 and D1S1631 was also identified as demonstrating possible linkage to the "low level of response to alcohol" phenotype with a maximum lod score of 2.0 [9]. Linkage was also supported in the COGA dataset of an endophenotype characterized by a later age of onset of regular drinking and higher harm avoidance to a region near D1S518 on 1q [10]. Mouse linkage analyses have also shed light on the genetic determinants of alcohol consumption. The extensive syntenic homology between the mouse and human genomes enables predictions about which human loci are syntenic with mouse alcohol related loci. Buck and co-workers, who studied several mouse alcohol related phenotypes, predicted that genes related to physical dependence on ethanol may localize to human chromosome regions 1q21-43, 2q11-32, 5p15, 5q14-21, and 9p24-22, 10q23-26 [11]. The purpose of the current study was to test the hypothesis that the positive linkage reported on human chromosome 1p21-23 by the COGA study and elsewhere on chromosome 1 could be replicated in a United Kingdom sample of families multiply affected by alcoholism.

## Results

Selection criteria for the families eliminated all but 9% of the families contacted from all sources. Press contacts were found to be the most productive source of suitable families willing to participate in the study, accounting for over 50% of subjects recruited. There appeared to be no bias in the affection status of subjects recruited from the different sources [12]. The affection status of all the subjects is summarized in table 1. The individuals interviewed were 297 and 176 subjects were genotyped for the purpose of this study. Alcohol consumption and severity of dependence indices measured using the severity of alcohol dependence questionnaire (SADQ) confirmed that ADS subjects were the most severely affected, the next level of lesser severity of alcoholism was defined by the Research Diagnostic Criteria of Alcoholism (RDCA) and then by the Heavy Drinking category (HD). The mean maximum regular lifetime consumption of the HD subjects (including the RDCA and ADS subjects, who are all HD) is 138 units/week. 80% of HD subjects reported a period of at least one month during which they were drinking in excess of 50 units/week for males or 35 units/week for females, which are the amounts above which the Royal College of Psychiatrists advises that drinking is likely to be harmful (Royal College of Psychiatrists, 1986). Almost half the HD subjects (49%) gave a history of a one month or longer period during which they were drinking in excess of 150 units/week for males, and 100 units/week for females. The mean SADQ score for ADS subjects was just below 30 (SD 14.2). The range for this rating scale was from 0 to 60. Stockwell and co-workers [13] suggested that scores above 30 indicate severe dependence. In our sample, 40% of the ADS subjects were severely dependent according to this definition.

**Table 1:** Proportion of the individuals for each affection status category and their mean age (*TT *Lifelong abstainers; *SD *Social Drinkers; *HD *Heavy drinkers; *RDCA *Research Diagnostic Criteria Alcoholism; *ADS *Alcohol Dependence Syndrome)

*Affection Status*	*Family Sample*		
	Number Genotyped	%	Mean age ± SD (yrs) in all cases and relatives

TT	7	4	58.2 ± 13
SD	43	24	50.4 ± 17.1
HD	22	13	41.5 ± 14.1
RDCA	50	28	41.5 ± 13.5
ADS	54	31	42.1 ± 11.9

### HD affection model

**Figure 1:**
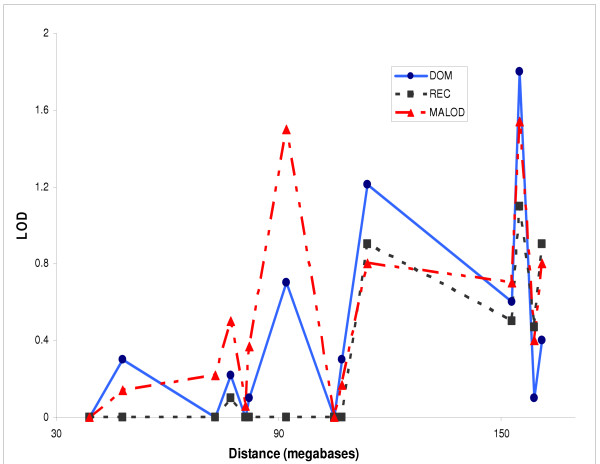
Two-point linkage analysis for the Heavy Drinking (HD) category under the dominant and the recessive model (HLOD) and model-free analysis (MALOD)

Assuming dominant transmission, two-point heterogeneity LODS (HLOD) for linkage to the HD affections status gave a score of 1.8 near the marker D1S1653. The three-point multipoint analysis produced an HLOD of 1.8 with the markers D1S1653 and D1S1677. Analyses with the same markers assuming recessive transmission produced an HLOD of 1.1 with a two-point analysis and an HLOD of 1.5 with a three-point analysis. The model-free (MFLINK) analysis produced an MLOD of 1.5 for both the two-point and three-point analyses. Three-point analyses of D1S1677 and D1S1679 produced an HLOD of 1.7 assuming recessive transmission and a MALOD of 1.3 for the model-free three point analysis. The marker D1S1675, on the short arm of chromosome 1, produced an HLOD of 1.2 with both a two-point analysis and a three-point analysis in combination with D1S3723 assuming dominant transmission, Model-free analyses of D1S1588 produced a MALOD of 1.5 and 1.2 using two-point and three-point analyses, respectively. Figure 1 shows the two-point linkage HLOD results for the HD category under both the dominant and the recessive model and also the model-free MFLINK analyses. The HD category is a cumulative definition which includes all the more severely affected categories for linkage analysis as well as those at the borderline of becoming alcoholic. The HD category is justified because drinking at this level is well recognised to be a predictor of future alcoholism, an observation that has also been incorporated in another genome linkage study of alcoholism which also employs a similar definition [14]. We have consistently used this affection status category in all our previous linkage analyses and it has not been applied in an arbitrary manner that could have inflated any lod scores.

### RDCA affection model

Assuming recessive transmission the marker D1S1679 was linked to RDC alcoholism with a two-point HLOD of 1.0 and a three-point HLOD of 1.6 with the markers D1S1677 and D1S1679. The MLOD two point MFLINK score with D1S1677 was 0.9 and this increased to 1.4 with a three-point model-free analysis.

**Figure 2:**
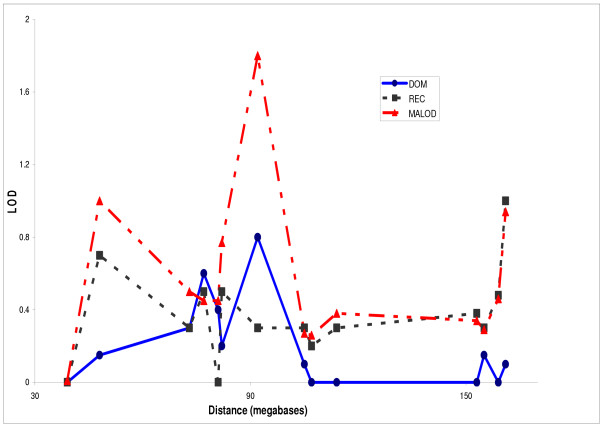
Two-point linkage analysis for the RDC alcoholism (RDCA) category under the dominant and the recessive model (HLOD) and model-free analysis (MALOD)

For the RDCA level of affection status the highest MALOD score of 1.8 was found near the telomere of chromosome 1p near the marker D1S1588 under the model free analysis. Figure 2 shows two-point linkage analysis for the RDCA category under the dominant and recessive models and also the model-free analyses.

### ADS affection model

The marker D1S1591 gave the highest maximum admixture lod (MALOD) of 1.00 with the model-free method of analysis for the ADS category. The results of MFLINK multipoint analysis for this and the other affection status models are shown in table 2.

**Table 2:** MFLINK two-point MALODs for the HD, RDCA and ADS diagnostic categories. In bold MALOD score value ≥1.

Markers	HD	RDCA	ADS
D1S1591	0	0	**1**
D1S2134	0.1	01	0.2
D1S1665	0.2	0.5	0
D1S532	0.5	0.4	0.4
D1S1728	0.1	0.4	0.4
D1S551	0.4	0.8	0.5
D1S1588	**1.5**	**1.8**	0
D1S1631	0	0.3	0.01
D1S3723	0.2	0.3	0.04
D1S1675	0.8	0.4	0.4
D1S1595	0.7	0.3	0.3
D1S1653	**1.5**	0.3	0.3
D1S1677	0.4	0.5	0.06
D1S1679	0.8	**1**	0.5

## Conclusion

The COGA study first reported evidence of a locus linked to alcoholism on chromosome 1p with the markers D1S532 and D1S1588 linked to Alcohol Dependence [6]. Curtis and coworkers, in their reanalysis of the initial COGA dataset computed a MALOD lod score of 1.75 with the marker D1S1588 [15]. The COGA group repeated the analysis in a new set of families and the lod score at D1S1588 was 1.6 in this replication data set. The combined analysis of the two COGA samples gave a lod of 2.6 [7]. The COGA study also conducted a regression method of linkage so as to include unaffected and discordant pairs. D1S1588 then gave a peak lod score of 2.9. Our own results of a lod of 1.8 at the region near D1S1588 offers support but not clear confirmation for the COGA findings. A second region on chromosome 1p, near D1S224, approximately 60 cM proximal to D1S1588, gave a multipoint lod score of 1.7 in the COGA study [6]. The current British study gave a MALOD score of 1.2 over this region with the marker D1S1675 under the HD model, and a MALOD of 1.2 with the marker D1S3723 for the RDCA category.

Dick et al [10] who analysed the COGA data have suggested that there is a locus on the long arm of chromosome 1 near the marker D1S518 linked to the endophenotype of a late age of onset of regular drinking and high harm avoidance. In a mouse recombinant inbred strain linkage study, a quantitative trait locus for physical dependence on alcohol was mapped to the murine chromosome 1 which is a region syntenic with human chromosome 1q21-43. In the present UK linkage analyses a three point HLOD score of 1.8 was found with the combined markers D1S1653 and D1S1677 for the HD phenotype. The positioning of the lod reported by Dick [10], the mouse genetic study and the UK linkage study are all compatible with a second locus for alcoholism which is on the proximal part of the long arm of chromosome 1 at 1q21.3-24.2.

In the UK linkage study the two regions on chromosome 1 are implicated with two different affection status categories. We could speculate that the two different loci on chromosome 1 could be mediating an effect on alcoholism through two loci that have different types of effect on susceptibility. The most interesting and probably important finding from the COGA linkage study is that the stratification of families that have members with both alcoholism and depression maximizes the lod to 5.12 on chromosome 1p near the markers D1S1648 and D1S1588 [14]. Without stratification based on clinical phenotype in the combined COGA sample a LOD score of 2.6 was obtained. These findings provide a strong indication that the 1p locus near D1S1588 is mediating its effect on alcoholism through a genetic susceptibility for depression and anxiety. A recent genome wide scan for quantitative-trait loci carried out by Fullerton and colleagues [16] identified a locus on chromosome 1p, D1S2868, linked to neuroticism. The authors speculated that this locus influences traits genetically related to neuroticism and maps near the locus, D1S1588, identified by Nurnberger et al. [14] for the endophenotype of alcoholism and depression. It is a generally accepted clinical fact that it is very difficult to diagnose whether depression or anxiety are predisposing to alcoholism or resulting from it. It is likely that both effects occur simultaneously. The twin and family study data to date, strongly supports the hypothesis that alcoholism both causes and augments pre-existing depression and anxiety [17-19]. The resolution of this issue at a biological and psychosocial level is a high priority for psychiatry as a whole because of the very high population prevalence of these comorbid disorders in most populations. The full understanding of these linkage findings in alcoholism may help us unravel basic aetiological mechanisms and help create new treatment and preventive strategies based on a fuller understanding of genetic and environmental factors involved in comorbidity. It would seem justified to start allelic association studies on chromosome 1 in order to fine map susceptibility genes for patients comorbid for depression, anxiety and alcoholism. A positive finding would gain support from the prior linkage studies of both alcoholism and affective disorders and vice versa. The presence of locus heterogeneity for genetic effects on depression, anxiety and alcoholism will require relatively large case-control studies of alcoholics with adequate clinical assessment to detect alcoholics with and without comorbid disorders.

## Methods

### a) Family Sample

Prior to commencing the study, ethical permission for this research project was obtained from the University College London Medical School Clinical Investigations Panel which has been updated in 2003 with multicentre research ethics committee (MREC) approval. Information stored on computer was registered under the 1984 Data Protection Act.

Caucasian families multiply affected by alcoholism, and suitable for linkage studies, were ascertained with the following selection criteria.

(a) Presence of two or preferably more cases of alcoholism as defined by the RDC.

(b) Large families, preferably with two or more generations willing and able to participate.

(c) Evidence of unilineal inheritance of alcoholism in the parental generation, which meant that families were excluded where both parents were affected.

(d) Willingness of as many family members as possible, especially affected individuals, to participate in the research project. The families were identified from hospital records, clinicians, advertisements in the media and other contacts [12]. The families were all white Caucasian in origin. The subjects were diagnosed using the following interview schedules: the Schedule for Affective Disorders and Schizophrenia – Lifetime version (SADS-L) which provides an RDC diagnosis of alcoholism [20], a "lifetime modification" of sections 1 and 3 of the Clinical Alcoholism Interview schedule [21], the Lifetime Drinking History [22] and a lifetime modification of the Severity of Alcohol Dependence Questionnaire (SADQ) [13]. Heavy Drinking (HD) was defined as drinking in excess of the Royal College of Psychiatrists recommendations, i.e. more than 14 units per week for females or 21 units per week for males, for over one month (Royal College of Psychiatrists, 1986). Diagnoses were made at three levels and these were employed in the linkage analyses in a hierarchical manner. All subjects fulfilling criteria for the Alcohol Dependence Syndrome (ADS) also fulfilled the criteria for RDC alcoholism (RDCA) and HD. All subjects meeting criteria for RDCA by definition also met the criteria for HD. Individuals who were drinking regularly below these limits, or who drank infrequently, were classified as social drinkers (SD), these individuals were considered as unaffected in the linkage analyses. Subjects who had never drunk alcohol, or had only one or two drinks in a lifetime, were considered to be lifelong abstainers (TT) and were also considered as unaffected. In order to check the RDCA, HD, SD and TT assessments, the interview schedules were examined by two independent psychiatrists. Where discrepancies arose between two raters, a consensus diagnosis was reached by process of joint discussion and consideration of the data.

### b) Laboratory procedures

Fifty nanograms of total genomic DNA was extracted from venous blood samples and amplified by Polymerase Chain Reaction (PCR) with oligonucleotide primers using standard methodology [23]. Short Tandem Repeat markers from the Research Genetics Set 9 genome screening panel and key markers from the COGA study were typed. A M13 tail was added to one of the oligonucleotide primers which was used to hybridise with a complementary oligonucleotide pre-labelled with infra red dyes that fluoresce at either 700 or 800 nm. The resulting images from argon laser scanning allow a highly detailed visualisation of genetic polymorphisms [24]. Gel electrophoresis and pattern visualization was performed using LI-COR Model 4200 automated fluorescent DNA sequencers [25].

Ten markers on 1p21-35 were genotyped: D1S1591, D1S2134, D1S1665, D1S1728, D1S532, D1S1588, D1S551, D1S1631, D1S3723 and D1S1675. Four markers on 1q were genotyped: D1S1595, D1S1653, D1S1677 and D1S1679. Marker order and intermarker distances were as compiled in the Ensembl database . Positions of the markers are shown in table 3.

**Table 3:** Marker location and type of polymorphism

**Markers**	**Mb**	**Type**
D1S1591	39	TETNUC
D1S2134	48	TETNUC
D1S1665	73	TETNUC
D1S532	77	TETNUC
D1S1728	81	TETNUC
D1S551	82	TETNUC
D1S1588	92	TRINUC
D1S1631	105	TRINUC
D1S3723	107	TETNUC
D1S1675	114	TETNUC
D1S1595	153	TETNUC
D1S1653	155	TETNUC
D1S1679	159	TETNUC
D1S1677	161	TETNUC

Genotypes were read blind to diagnostic information. Tests for Mendelian inheritance of marker data were performed and inconsistent genotypes were repeated or omitted.

### c) Linkage analysis

The linkage analysis was performed using the classical lod score method and using likelihood-based model-free analysis carried out with the MFLINK program [26]. For lod score analyses, the programs MLINK and LINKMAP were used from the FASTLINK package [27]. Three affection models were used: ADS, RDCA and HD. Each affection model was analysed assuming dominant transmission, recessive transmission and using the model-free method. For dominant models the frequency of the abnormal allele was set to 0.02 and for recessive models to 0.2. The penetrance for normal and abnormal genotypes respectively were set to 0.02 and 0.5 for ADS, to 0.04 and 0.7 for RDCA and to 0.2 and 0.9 for HD. These penetrance values were chosen to produce models approximately consistent with prevalence data from previous epidemiological research of 0.04, 0.06 and 0.2 for ADS, RDCA and HD respectively [28,29]. Two-point and three-point analysis was carried out for all affection definitions and transmission models. Classical linkage analysis was carried out under the assumption that locus heterogeneity might be present, yielding an HLOD statistic.

## Authors' contributions

IG carried out the genotyping and produced the manuscript

CCHC collected the multiplex family sample and participated in the design of the study

WK participated in genotyping

AD participated in genotyping

AM participated in genotyping

DC performed the linkage analysis

HMDG conceived the study and participated in its design and coordination

All authors read and approved the final manuscript
